# CD34 Promotes Pathological Epi-Retinal Neovascularization in a Mouse Model of Oxygen-Induced Retinopathy

**DOI:** 10.1371/journal.pone.0157902

**Published:** 2016-06-28

**Authors:** Martin J. Siemerink, Michael R. Hughes, Marchien G. Dallinga, Tomek Gora, Jessica Cait, Ilse M. C. Vogels, Bahar Yetin-Arik, Cornelis J. F. Van Noorden, Ingeborg Klaassen, Kelly M. McNagny, Reinier O. Schlingemann

**Affiliations:** 1 Ocular Angiogenesis Group, University of Amsterdam, Academic Medical Center, Amsterdam, The Netherlands; 2 Department of Ophthalmology, University of Amsterdam, Academic Medical Center, Amsterdam, The Netherlands; 3 Department of Cell Biology and Histology, University of Amsterdam, Academic Medical Center, Amsterdam, The Netherlands; 4 Department of Medical Genetics, University of British Columbia, Vancouver, BC, Canada; 5 The Biomedical Research Centre, University of British Columbia, Vancouver, BC, Canada; 6 Department of Experimental Medicine, University of British Columbia, Vancouver, BC, Canada; Indiana University College of Medicine, UNITED STATES

## Abstract

The sialomucins CD34 and podocalyxin (PODXL) are anti-adhesive molecules expressed at the luminal membrane of endothelial cells of small blood vessels and facilitate vascular lumen formation in the developing mouse aorta. CD34 transcript and protein levels are increased during human angiogenesis, its expression is particularly enriched on endothelial tip cell filopodia and CD34 is a marker for tip cells *in vitro*. Here, we investigated whether CD34 merely marks endothelial tip cells or has a functional role in tip cells and angiogenesis. We assessed that silencing CD34 in human microvascular endothelial cells has little effect on endothelial cell migration or invasion, but has a significant effect on vascular-endothelial growth factor-induced angiogenic sprouting activity *in vitro*. *In vivo*, the absence of CD34 reduced the density of filopodia on retinal endothelial tip cells in neonatal mice, but did not influence the overall architecture of the retinal vascular network. In oxygen-induced retinopathy, *Cd34*^-/-^ mice showed normal intra-retinal regenerative angiogenesis but the number of pathological epi-retinal neovascular tufts were reduced. We conclude that CD34 is not essential for developmental vascularization in the retina, but its expression promotes the formation of pathological, invasive vessels during neovascularization.

## Introduction

Angiogenesis is the process by which vessels grow and branch from existing vessels to generate new vascular beds (reviewed in ref [1]). Physiological angiogenesis in adults is critical for wound healing, reproduction, and tissue growth and regeneration. Accordingly, insufficient or pathological angiogenesis underlies many human diseases including myocardial infarction, delayed wound healing (ulcerative disease), neurological disease, impairment of vision (proliferative retinopathies) and tumor growth and metastasis (reviewed in ref [2]). Thus, a more complete understanding of the mechanisms regulating both physiological and pathological angiogenesis may have clinical implications for the treatment of disease and advancement of regenerative therapies.

The highly organized vascular sprouts in angiogenesis are composed of distinctly differentiated endothelial cell subtypes that act in a strict hierarchical fashion [3, 4]. Among these subtypes are endothelial tip cells, the leading cells of vascular sprouts that form filopodia to aid migration towards a source of growth factors such as vascular endothelial growth factor-A (VEGF-A); and, to direct adjacent endothelial cells to form and elongate the stalk of sprouting vessels [4–8]. In addition, tip cells have other functions that are essential for capillary sprouting such as a specific proteolytic machinery that is required for migration and invasion into the extracellular matrix or ischemic areas [9, 10]. Whether tip cell functions differ in sprouting angiogenesis under normal conditions and pathological conditions is unknown.

CD34 and podocalyxin (PODXL) are transmembrane anti-adhesive sialomucins that are ubiquitously expressed on the luminal surface of endothelial cells in capillaries [11–15]. CD34 and PODXL also act as anti-adhesive molecules during lumen formation in the developing mouse aorta by maintaining or promoting the separation between contralateral apical endothelial cell lumen surfaces [16]. It has been shown in *Cd34* knockout (*Cd34*^-/-^) mice that, when expressed on blood cells, CD34 enhances the adhesion, mobility and invasiveness of hematopoietic progenitors, mast cells, and eosinophils [17–20]. High expression of CD34, but not PODXL, on endothelial tip cells and their filopodia [4, 21] suggests that CD34 may play a role in angiogenesis that is specifically related to filopodia functions or architecture [7, 13, 22, 23]. A role of CD34 in tumor angiogenesis was suggested by the observation that *Cd34* deletion in mice (specifically in non-hematopoietic lineages) impaired early tumor growth due to a delay in angiogenesis [24]. Whether this effect is specifically related to expression of CD34 on tip cells is not known. We have found that only a subset (approximately 10%) of cultured human umbilical vein endothelial cells (HUVECs) express CD34 and this CD34-positive population has a distinct endothelial tip cell phenotype [21]. We also showed that this subpopulation of CD34^+^ tip cells is actively restored when isolated CD34^-^ HUVECs are re-cultured [21]. Furthermore, stimulation of HUVECs with angiogenic growth factors such as VEGF-A induced CD34 expression [25]. Therefore, high CD34 expression marks endothelial cells with tip cell activity *in vitro*. However, it is not known whether CD34 has a functional role in tip cell behavior or angiogenesis in general.

In the following study, we investigated the role of CD34 in angiogenesis using *in vitro* angiogenesis models in the presence or absence of CD34-specific small interfering RNA (siRNA) and in physiological and pathological angiogenesis *in vivo* using *Cd34*^-/-^ mice. In a model of oxygen-induced retinopathy (OIR), we provide evidence that, although CD34 is not necessary for physiological retinal blood vessel development, it promotes epi-retinal tuft formation in pathological retinal angiogenesis.

## Materials and Methods

### Cells and cell cultures

Immortalized human microvascular dermal endothelial (HMEC-1) cells were grown on culture flasks coated with 2% gelatin containing M199 medium (Gibco, Grand Island, NY, USA) supplemented with 5% human serum (Academisch Medisch Centrum Hospital, Amsterdam, The Netherlands), 5% fetal bovine serum (FBS) (Biowhittaker, Walkersvillle, MD, USA) and 1% penicillin-streptomycin-glutamine (Gibco). HMEC-1 cells between passages 30 and 40 were used for all experiments. All cells were grown in a humidified 37°C incubator with 5% CO_2_.

### Small interfering RNA (siRNA) transfection

HMEC-1 were transfected with either CD34-specific siRNA pool or non-targeting siRNA (Accell SMARTpool, Dharmacon, Lafayette, CA, USA). The siRNAs were transfected using the reverse-transfection method, according to the manufacturer’s protocol. Cells were either collected at 72 h after transfection for RNA or spheroids, formed 52 h after transfection, were embedded in collagen gels (72 h after transfection) for sprouting assays.

### FACS and flow cytometry

For flow cytometric analysis, HMEC-1 were labeled with anti-human podocalyxin antibody (goat pAb IgG, AF1556, R&D Systems, Minneapolis, MN, USA), anti-human CD34 antibody (mouse mAb IgG1, QBend10 clone, R&D Systems) or the appropriate isotype control antibody at the same concentrations followed by a fluorchrome-conjugated secondary antibody (Alexa Fluor® 647 chicken anti-goat IgG, A21469; ThermoFisher Scientific, Waltham, MA, USA) or fluorescein isothiocyanate (FITC) goat anti-mouse IgG (1010–02; Southern Biotech, Birmingham, AL, USA). Data was collected on an LSR II instrument (Becton Dickinson (BD), Mountain View, CA, USA) and analyzed using FlowJo v10.0.08 software (FlowJo, Ashland, OR, USA). For cell-sorting, HMEC-1 were labeled with anti-CD34 antibody (QBend10 clone; Sanquin, Amsterdam, The Netherlands) and sorted for CD34 expression on a FACSAria or FACSCanto-II instrument (Becton Dickinson, Mountain View, CA, USA) as described previously [21].

### RNA isolation and gene expression analysis

Total RNA was isolated from cells using the TRIzol method (Invitrogen, Carlsbad, CA, USA). Approximately 1 μg of total RNA was used for DNAse treatment (amplification grade; Invitrogen) and reverse transcription into first strand cDNA using Superscript III and oligo(dT)_12–18_ (Invitrogen). The primers used for the CD34 quantitative PCR (qPCR) analysis were 5’-GGAGCAGGCTGATGCTGATG-3’ (For); 5’-ATCCCCAGCTTTTTCAGGTCAGAT-3’ (Rev). NCBI BLAST confirmed specificity of the primers. The presence of a single PCR product was verified by both the presence of a single melting temperature peak and detection of a single band of the expected size on agarose gels. Non-template controls were included to verify the method and the specificity of the primers. Mean primer efficiency was 96% ± 3%. Real-time qPCR was performed as described previously [26], using a CFX96 real-time PCR detection system (Bio-Rad Laboratories, Hercules, CA, USA).

### Spheroid-based angiogenesis model

Endothelial cells (750 cells/spheroid) were seeded in medium containing methylcellulose (Sigma-Aldrich, St Louis, MO, USA) to form spheroids [27]. After 24 h, cells were embedded in collagen gel in the presence or absence of 50 ng/ml human recombinant VEGF-A (Sanquin) and allowed to sprout for 24 h. At least 8 spheroids per group were analyzed under an inverted microscope (Leica Microsystems, Mannheim, Germany) and phase contrast images were quantified using image analysis and ImageJ software [28].

### Scratch assay

HMEC-1 were transfected with siRNAs as described above and cultured in 12-well tissue culture plates until confluent. The confluent monolayer was scraped with a 200-μl pipette tip to generate a wound and was rinsed twice with medium. Micrographs were taken at 40× magnification using an inverted microscope (Leica Microsystems) and phase contrast images were quantified using image analysis and ImageJ software [28].

### Cell invasion assay

HMEC-1 were transfected with siRNAs as described above and seeded (5×10^4^ cells per well) on transwell Boyden chamber inserts (8 μm pores; Corning, Lowell, MA, USA) containing a polycarbonate filter as previously described [29]. Filters were pre-coated with Matrigel (BD Discovery Labware, Bedford, MA, USA) diluted 1:3 in M199 basal medium to create an artificial internal limiting membrane. Inserts containing cells were placed in M199 supplemented with 2% human serum in 24-well plates. HMEC-1 migration was stimulated by adding complete medium to the lower well of the Boyden chamber. After 24 h, membranes were washed with ice-cold phosphate-buffered saline (PBS) (Lonza, Walkersville, MD, USA) and the upper surface of the insert was swabbed to remove non-migrated cells. Cells that had migrated through the pores of the filter were either fixed in 4% paraformaldehyde (Electron Microscopy Sciences (EMS), Hatfield, PA, USA) and stained with Hoechst or fixed with absolute methanol and stained with Giemsa. Migration was evaluated as the mean number of migrated cells in 5 high-power fields (HPF) per well (20× magnification). Each condition was assayed in triplicate and each experiment was performed at least twice.

### Mice

All mice used in this study were backcrossed to C57Bl/6J mice for more than 12 generations. *Cd34*^-/-^ mice were generated as described previously [30]. Mice were bred and maintained in specific pathogen-free conditions at the Biomedical Research Centre (The University of British Columbia (UBC), Vancouver, BC, Canada). All animal experiments were approved by UBC’s animal care committee and were conducted humanely following institutional and Canadian Council on Animal Care guidelines (Protocol #A11-0289). Mice were sacrificed humanely by carbon dioxide inhalation at a flow rate of 3 litres/min (20–30% chamber volume/min) as per UBC ACC SOP.

### Oxygen-induced retinopathy model and analysis of postnatal retinal angiogenesis

The OIR model was carried out as previously described [31] with the use of a BioSpherix ProOX A chamber equipped with ProOx P110 oxygen controller (BioSpherix, Lacona, NY, USA). Postnatal day 7 (P7) pups together with their nursing dams were placed for 5 consecutive days in a 75% oxygen chamber. Litters of 8 pups or less had 1 nursing dam; those with more than 8 pups had 2 nursing dams. The chamber was only opened briefly between P7 and P12 when the nursing dams were replaced with foster dams to mitigate any adverse effects of hyperoxia on the nursing dams. At P12, the pups were returned to room air (21% oxygen). Pups were sacrificed and their eyes were collected at P12, P17 and P21. In addition, mouse eyes were collected from pups raised in room air at P1, P3, P5, P7, P9 and P25 as controls.

Eyes were fixed for 30 min in 4% paraformaldehyde prepared in PBS. Retinas were dissected, fixed overnight in 4% paraformaldehyde at 4°C, dehydrated in methanol and stored in methanol at -20°C. Before immunofluorescence analysis, retinal whole-mounts were rehydrated, permeabilized in PBS containing 1% bovine serum albumin (BSA) (Sigma-Aldrich) and 0.5% Triton X-100 (Sigma-Aldrich) at 4°C overnight and washed with PBS. Retinal whole-mounts were blocked in PBlec (PBS (pH 6.8) with 1% Triton X-100, 0.1 mM CaCl_2_, 0.1 mM MgCl_2_, 0.1 mM MnCl_2_ (all from Sigma-Aldrich)), and incubated with AF488-labeled or AF594-labeled lectin from *Bandeiraea simplicifolia* (isolectin B4) (Invitrogen) in PBlec at 4°C overnight. After extensive washing in PBS, the retinas were either flat mounted in Vectashield (Vector, Burlingame, CA, USA) or processed for multiple labeling, using primary antibodies directed against mouse CD34 (clone RAM34; eBioscience, San Diego, CA, USA) or mouse PODXL (clone 192704, R&D Systems). Secondary antibodies used were cyanine-3 (Cy3)-labeled donkey anti-goat and Cy3-labeled goat anti-rat, respectively (Jackson ImmunoResearch Laboratories Inc, West Grove, PA, USA). Images were taken using a wide-field fluorescence microscope or confocal microscope (Leica Microsystems). In control eyes, filopodia at the vascular front were analyzed in 20 microscopic fields selected randomly from 5 retinas per group of mice (mutants or wild type littermates) and quantified using image analysis and ImageJ software [28]. For the OIR model, the retinal avascular areas and neovascularization areas were quantified using Adobe Photoshop CS4 software according to a published protocol [28, 32].

### Statistical analysis

Values are given as mean values ± SD or SEM, as indicated. Data are represented as averages of independent experiments, performed in duplicate or triplicate. Statistical analyses were performed using the Student’s t-test or two-way ANOVA and P-values < 0.05 were considered to indicate statistically significant differences.

## Results

### CD34 marks VEGF-responsive endothelial tip cells

**Fig 1:**
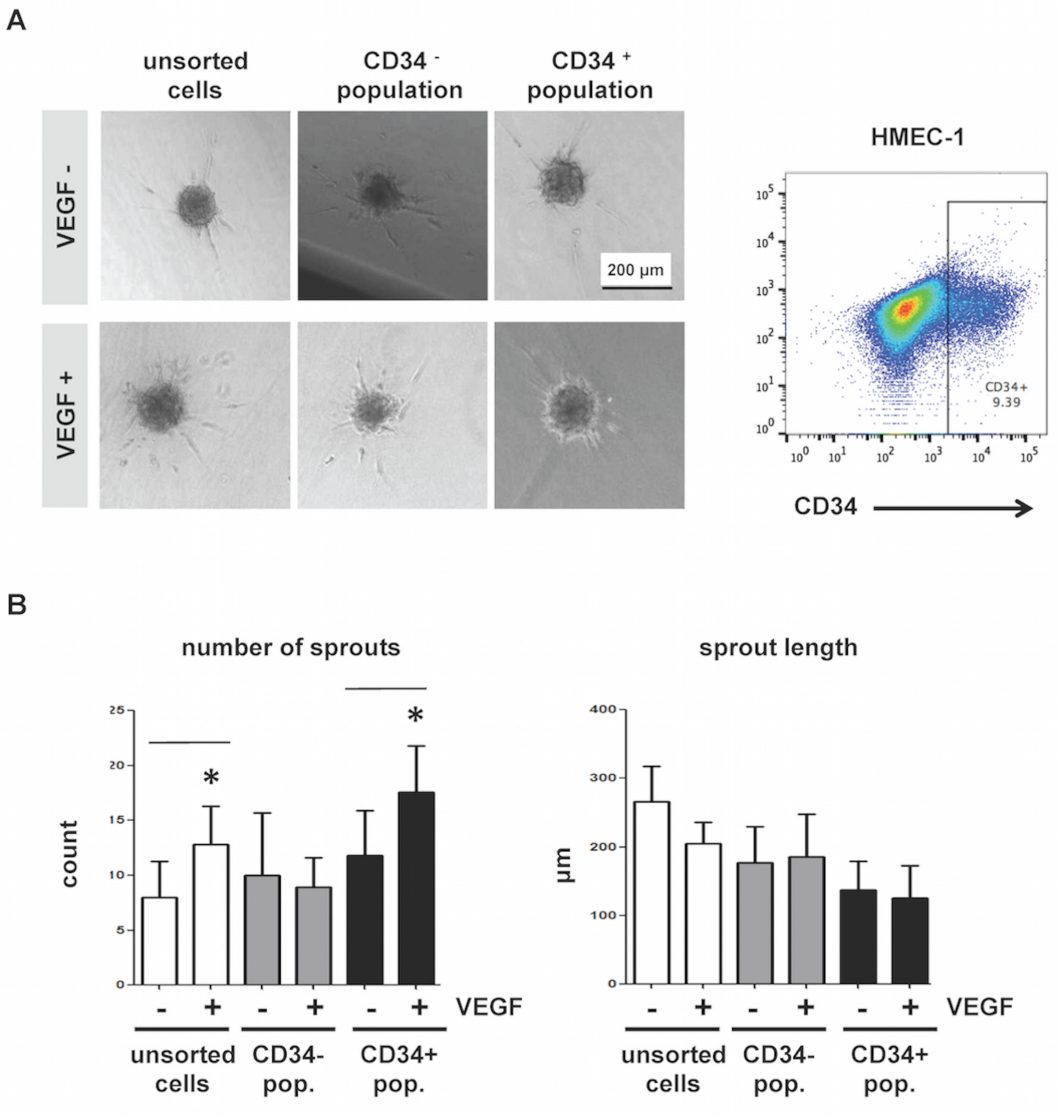
The CD34^+^ fraction of HMEC-1 cultures contains the VEGF-induced angiogenic sprouting activity. (A, left panel) Representative images of spheroids that were generated from HMEC-1 were either unsorted or FACS-sorted, based on CD34 cell surface expression (CD34^-^ and CD34^+^ populations). Spheroids were embedded in collagen gel supplemented with (**+**) or without (-) VEGF. Scale bar = 200 μm. (A, right panel) Flow cytometry dot plot demonstrating gating for HMEC-1 sorting based on CD34 expression. (B) The number of sprouts per spheroid and the mean sprout length were quantified using Image J. Error bars represent standard deviation. *Significantly different from unstimulated control (VEGF-) with P < 0.05.

We previously reported that CD34 marks cells with an endothelial “tip cell” phenotype and gene expression pattern in cultures of HUVEC [21]. To evaluate a possible functional role of CD34 in endothelial cells during angiogenesis, we carried out *in vitro* experiments using HMEC-1. Similar to HUVEC [21], approximately 9–10% of HMEC-1 express high levels of CD34 (Fig 1A, right panel). To test the functional contribution of CD34^+^ and CD34^-^ cells to sprouting, we generated spheroids of FACS-isolated populations of CD34^+^ or CD34^-^ HMEC-1 and embedded them in collagen gels in the presence or absence of VEGF-A. After 24 h, CD34^+^ sorted HMEC-1 showed a significant increase in the number of sprouts (but not sprout length) in response to VEGF-A, as did spheroids composed of unsorted HMEC-1 cells. The CD34^-^ HMEC-1 spheroids were unresponsive to VEGF-A (Fig 1A and 1B). This shows that VEGF-A-responsive angiogenic sprouting activity in HMEC-1 cultures is associated with the CD34^+^ population.

### CD34 promotes VEGF-induced angiogenic sprouting *in vitro*

**Fig 2:**
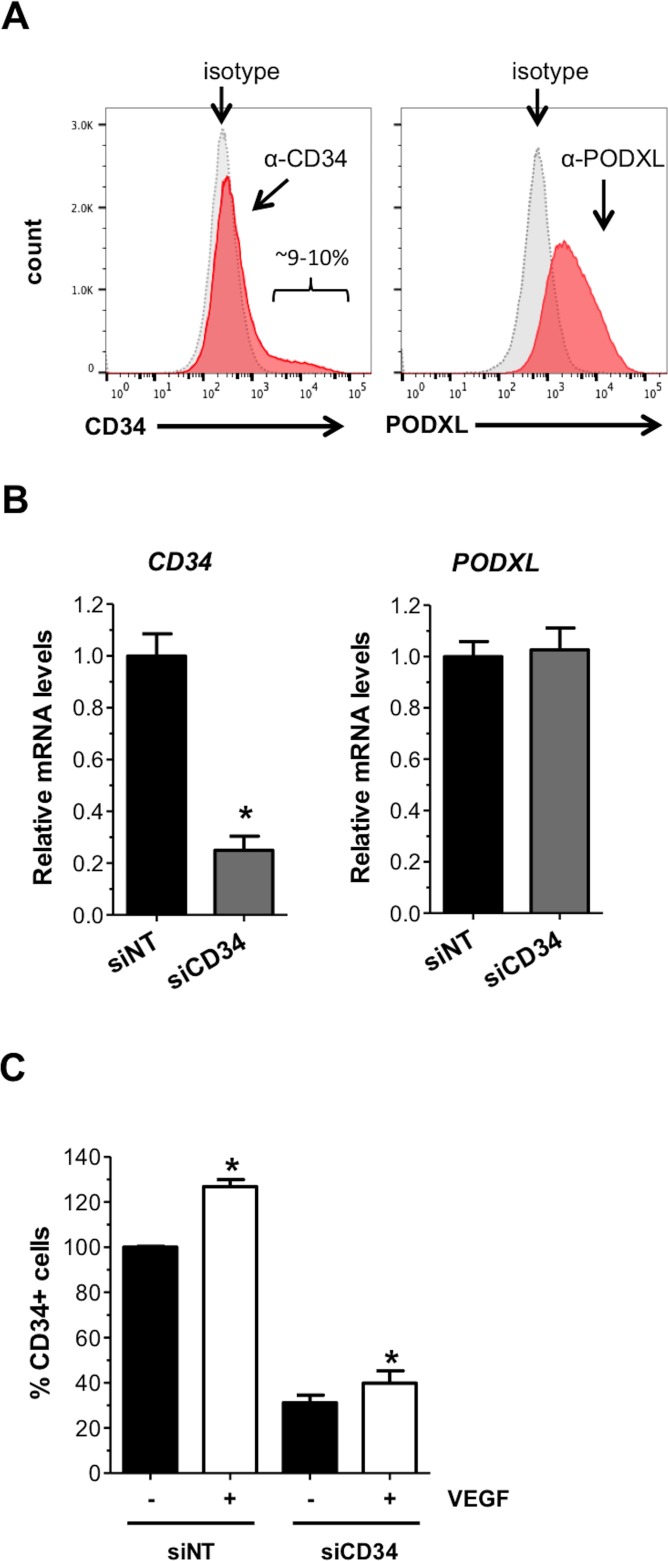
Silencing of CD34 expression in HMEC-1. (A) Representative histograms showing the intensity of anti-CD34 (left panel) or anti-podocalyxin (right panel) labeling of parental HMEC-1 compared to an isotype control (grey peak). (B) Quantitative PCR (qPCR) analysis of CD34 and PODXL mRNA levels and (C) flow cytometric analysis of CD34 protein expression on the membrane of HMEC-1 72 h after transfection with CD34-targeting (siCD34) or non-targeting (NT) (siNT) siRNA. Stimulation with VEGF induced CD34 plasma membrane expression in both siNT-treated and siCD34 treated HMEC-1. However, CD34 expression in siCD34-treated HMEC-1 was less than 30% as compared to expression levels in siNT-treated cells. *Significantly different from non-stimulated cells with P < 0.05.

Although VEGF-A induced sprouting activity is associated with the CD34-expressing HMEC-1 population, it is possible that CD34 is simply a marker of “tip” like cells but does not serve a functional role. Therefore, to further investigate a possible functional role of CD34 in HMEC-1 sprouting angiogenesis or migration activity *in vitro*, we silenced CD34 expression in HMEC-1 using siRNA. Although only 10% of HMEC-1 in culture is positive for CD34, all cells express the closely related sialomucin podocalyxin (PODXL) (Fig 2A). Both CD34 and PODXL are widely expressed at the luminal plasma membrane of vascular endothelial cells. Knockdown of CD34 by siCD34 was confirmed at the mRNA transcript level by qPCR (Fig 2B, left panel). Notably, knockdown of CD34 did not alter gene expression of *PODXL* (Fig 2B, right panel). Flow cytometric analysis confirmed suppression of CD34 protein expression on the plasma membrane in the presence or absence of exogenous VEGF-A (Fig 2C).

**Fig 3:**
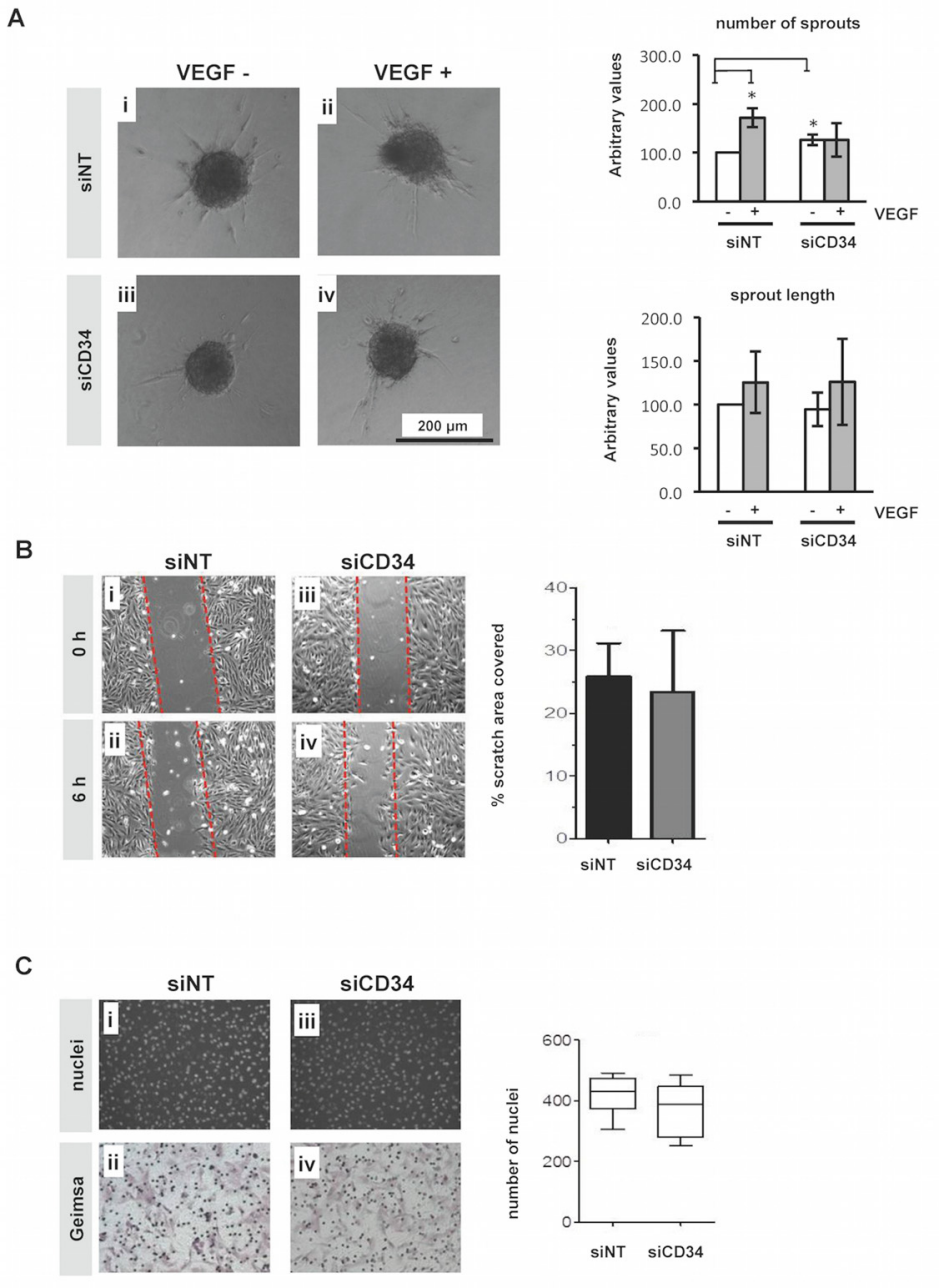
Effect of CD34 silencing on sprouting, cell migration and invasion. (A, left panel) Representative images of spheroids that were generated from HMEC-1 transfected with either a non-targeting siRNA (siNT) or siCD34 and subsequently embedded in collagen gel in the presence or absence of VEGF-A. (A, right panel) Spheroids were analyzed at 24 h after embedding and the number of sprouts per spheroid and average sprout length were quantified using Image J. Results were expressed relative to values of siNT transfected cells without VEGF. (B, left panel) siNT- and siCD34-transfected HMEC-1 were grown until confluent. Scratches were made using a pipette tip and images were taken at 0 and 6 h after scratching. (B, right panel) The percentage of width of the scratch filled with cells was quantified over time. (C, left panel) Representative images of HMEC-1 transfected with siNT or siCD34, located at the lower side of the Boyden filter after invasion through Matrigel visualized by DNA staining (i,iii) or Giemsa (ii,iv) staining at 20 h after seeding. (C, right panel) Cells invading the Matrigel were quantified by counting the number of nuclei per microscopic field. Error bars represent standard deviation, except for Fig 3C (right panel) where they represent 95% confidence interval; *Significantly different from siNT control with P < 0.05.

To explore whether CD34 gene ablation affects endothelial cell sprouting, migration and invasion, we performed three separate *in vitro* assays. First, we repeated the spheroid-based sprouting assay as described in Fig 1 using siCD34 transfected HMEC-1. Silencing of CD34 expression increased the number of sprouts per spheroid marginally (1.3-fold) in the absence of exogenous VEGF-A. However, spheroids in which CD34 was silenced did not respond to VEGF-A stimulation (Fig 3A). No significant differences were observed for sprout length when comparing spheroids silenced for CD34 with control spheroids (Fig 3A) and the results are in line with spheroids of FACS-isolated populations of CD34^+^ or CD34^-^ HMEC-1. Second, we performed a wound closure (scratch) assay using siCD34 transfected HMEC-1. This experiment showed that silencing of CD34 did not alter HMEC-1 migration (Fig 3B). Third, we seeded siCD34 transfected HMEC-1 into Boyden chambers containing membranes pre-coated with Matrigel to mimic the extracellular matrix of endothelium. Silencing of CD34 did not affect the level of HMEC-1 invasion after 20 hours in this assay (Fig 3C). Thus, although silencing of CD34 results in a marginal increase in spheroids sprouting, the most important result seems to be that HMEC-1 without CD34 expression do not respond to VEGF-A, although a basic level of sprouting is still intact. Migration and invasion of HMEC-1 were not affected by silencing of CD34.

### CD34 is expressed on endothelial tip cells and tip cell filopodia *in vivo*

Since our *in vitro* assays suggested that CD34 has a role in regulating VEGF-induced sprouting activity, we next wished to determine whether CD34 has a role in sprouting angiogenesis *in vivo*. In retinal angiogenesis, blood vessels are formed in an organized and directional manner and offer a controlled and physiological model to study angiogenesis *in vivo*. Our first objective was to determine the expression pattern of CD34 in the mouse retina during postnatal vessel development.

**Fig 4:**
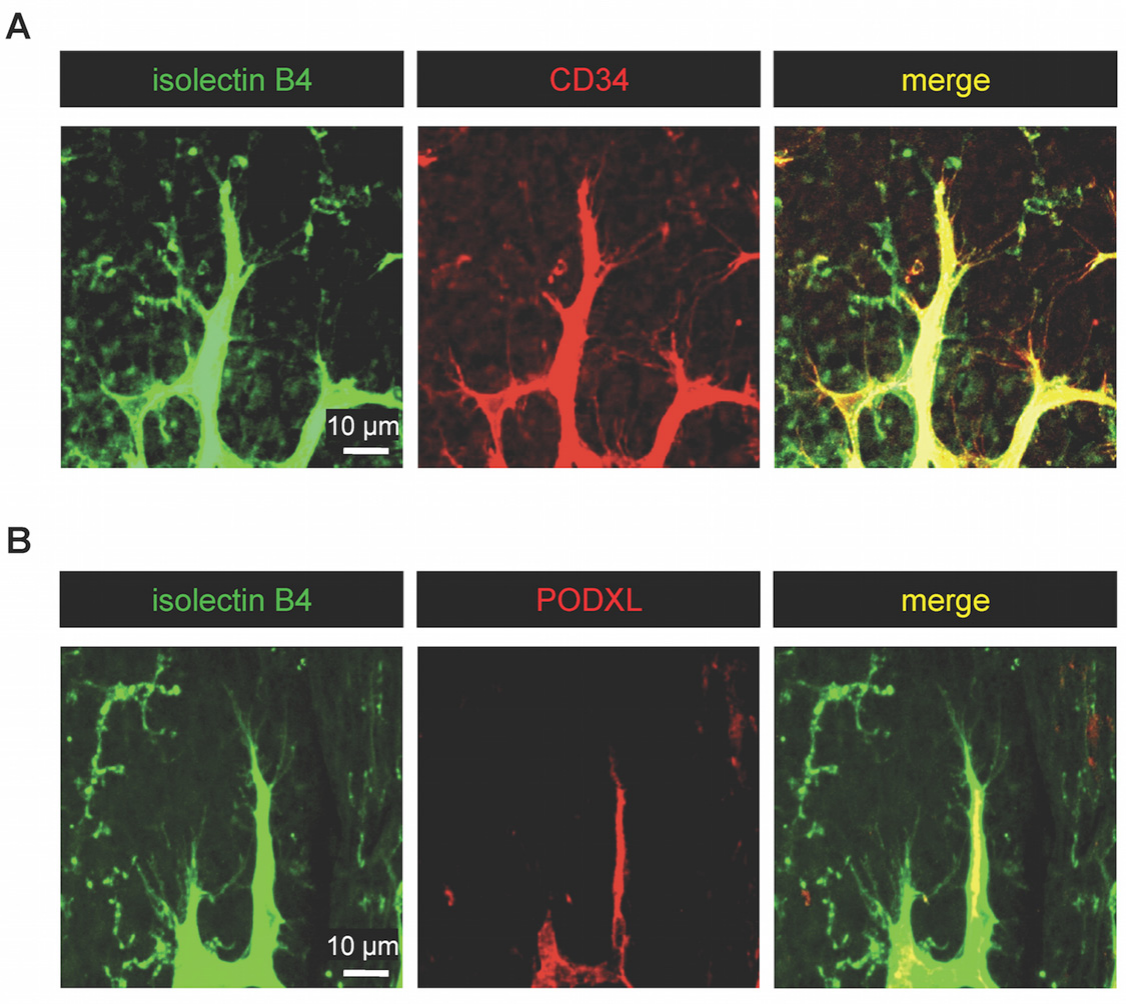
Endothelial tip cell filopodia in developing retinal vessels express CD34 but not podocalyxin. Immunofluorescence staining of retinal whole-mounts of wild type (Wt) mice at P5. Both CD34 and podocalyxin (PODXL) are expressed on developing vasculature. CD34 but not podocalyxin is expressed on endothelial tip cell filopodia. Isolectin B4 staining (green) was used to visualize tip cell filopodia. Scale bar = 10 μm.

Immunohistochemistry of whole-mount retinas harvested from P5 mouse pups showed that CD34 and PODXL are expressed on the vasculature in the developing retina. Although CD34 expression is limited to a select population in HUVEC and HMEC-1 cultures, CD34 is more widely expressed throughout microvasculature in humans and is expressed on filopodial of angiogenic tip cells during active angiogenesis [21]. Likewise, we found that in the developing retinal vessels of neonatal mice, CD34 is expressed on the filopodial extensions of tip cells at the angiogenic vessel front in addition to the expression throughout the advancing sprouts, phalanx and the lumen of the vessel stalk (Fig 4A). The distribution pattern of CD34 on endothelial cells in angiogenic tissues was similar to that of isolectin B4 (an endothelial marker used to visualize endothelial tip cells and their filopodia) (Fig 4A) [6, 7, 33–36]. Notably, PODXL is expressed only within the stalk (and phalanx) region of vessels and is absent from the filopodia (Fig 4B). These findings suggest that CD34 and PODXL have non-redundant functions in vascular endothelia.

### CD34 deletion reduces the number of tip cell filopodia without perturbing retinal vasculature development

**Fig 5:**
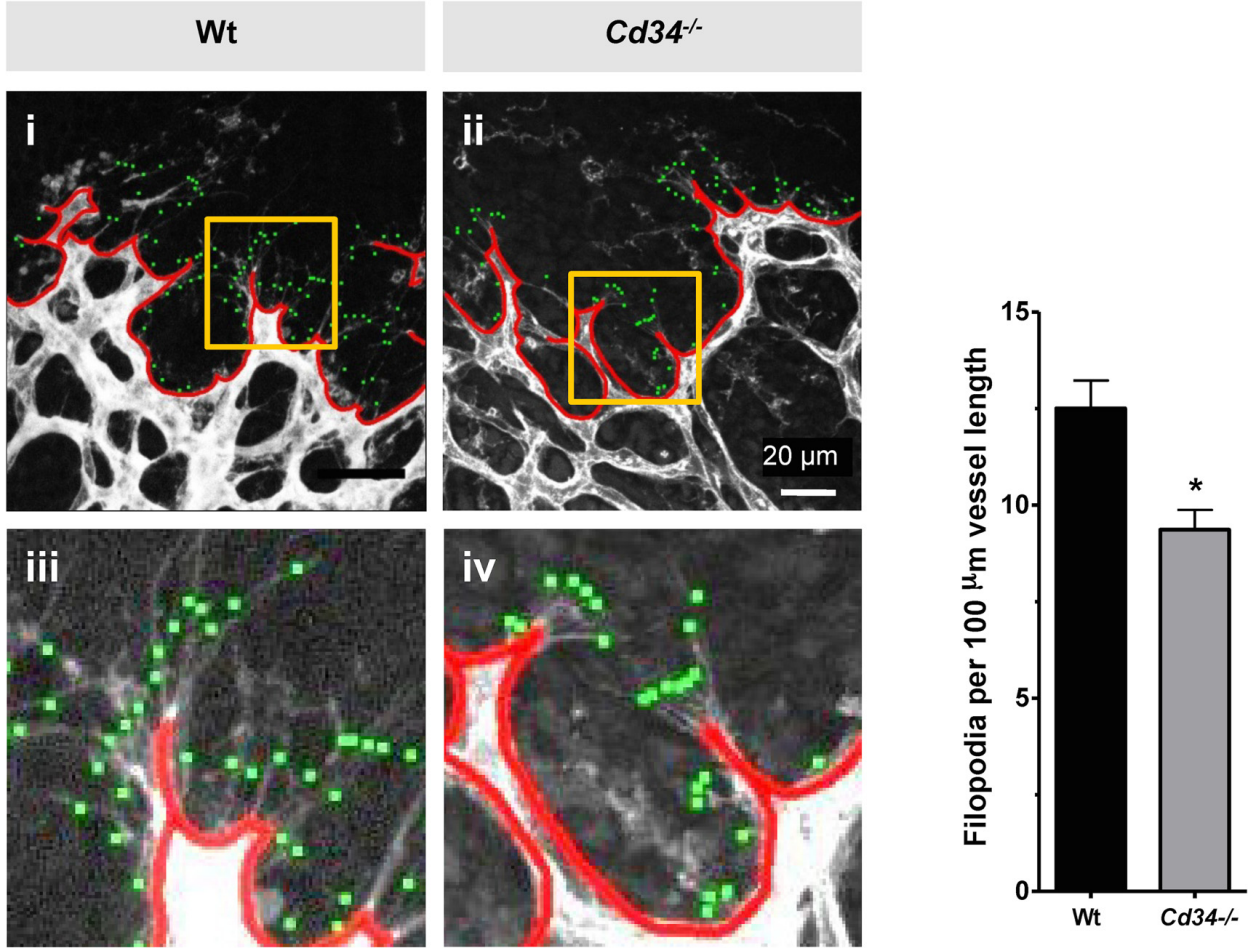
CD34 deletion reduces the number of endothelial tip cell filopodia. (A) Whole-mount isolectin B4 staining showing the angiogenic front of wild type (Wt) (i,iii) and *Cd34*^-/-^(ii,iv) mouse retinas at P5. Scale bar (i,ii) = 20 μm. Tip cell filopodia were marked (green dots) and normalized to vessel length (red lines, see insets iii, iv). (A, right panel) Quantification of filopodia per 100 μm vessel length. Error bars represent standard errors of the mean. * Significantly different from wild type with P < 0.05.

Since CD34 is an anti-adhesive molecule, we hypothesized that CD34 reduces adhesion of filopodia to the extracellular matrix or membranes of other cells, thereby facilitating extension of tip cell filopodia. To test this hypothesis, we examined the morphologic features of tip cells during retinal development under normoxic conditions in wild type and *Cd34*^-/-^ mice. Detailed analysis of the vascular front revealed that filopodia density was reduced by 30% in *Cd34*^-/-^ mice at P5 as compared to wild type mice (Fig 5).

**Fig 6:**
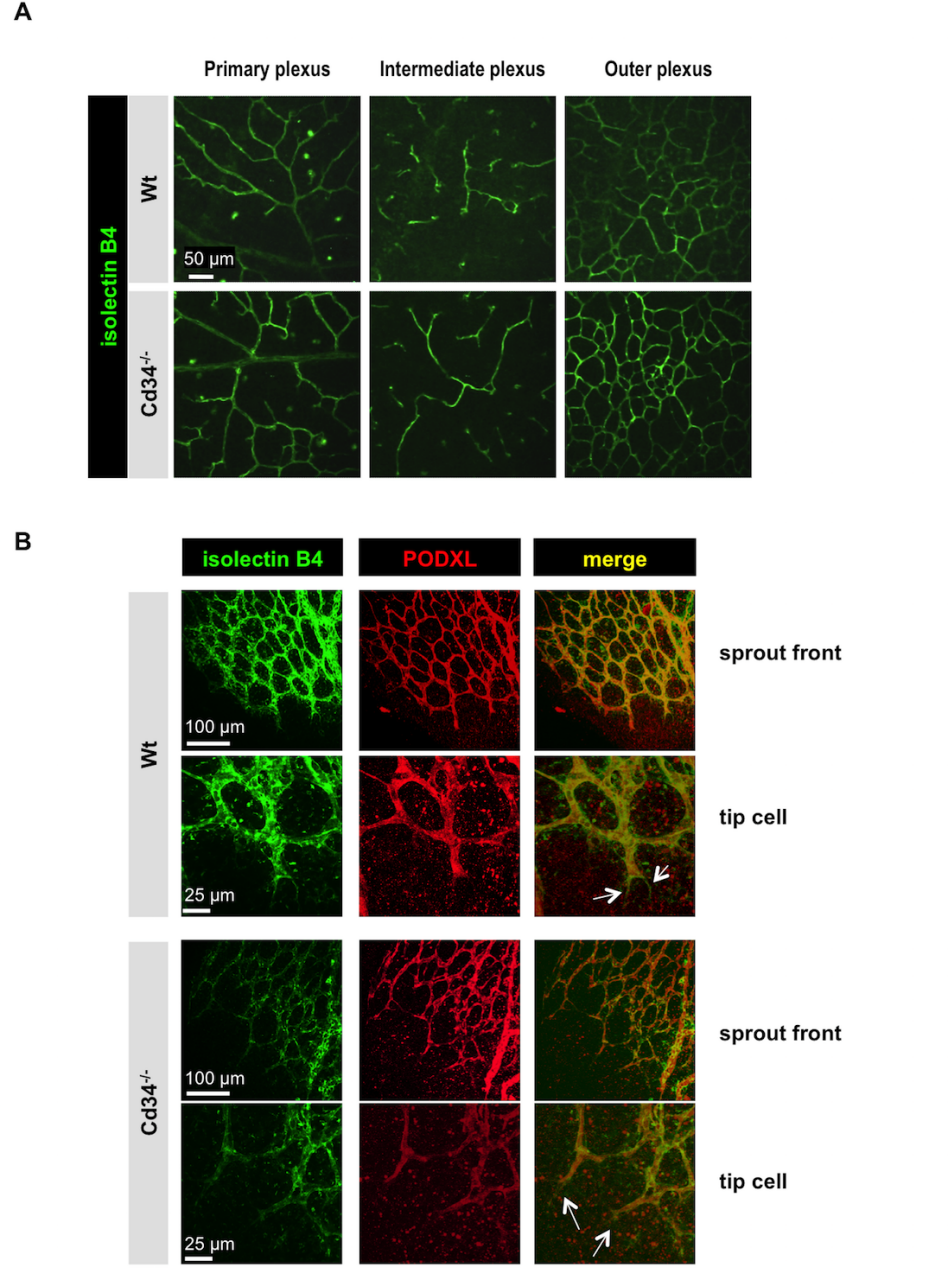
Retinal vessels develop normally in *Cd34*^-/-^ mice under normoxic conditions. (A) Isolectin B4 staining (green) of retinal whole-mounts of wild type (Wt) and *Cd34*^-/-^ mice under normoxic conditions at P25. The plexuses of retinal vessels in both the inner and outer retina are similar in Wt and *Cd34*^-/-^ mice at P25. Scale bar = 50 μm. (B) Immunofluorescence staining of podocalyxin (PODXL, red) and isolectin B4 (green) of whole-mounts of retinas harvested from wild type (Wt) and *Cd34*^-/-^ mice at P5. The arrows in the merge (yellow) indicate tip cell filopodia that stain with isolectin B4 but do not co-express podocalyxin. Podocalyxin is not expressed on endothelial tip cell filopodia in either wild type (Wt) or *Cd34*^-/-^ mice. Scale bars = 100 μm (sprout front) or 25 μm (tip cell).

To determine whether the reduction density of filopodia in *Cd34*^-/-^ mice has consequences for the physiological development of retinal vessels, we studied the retinal vasculature in normoxic wild type and *Cd34*^-/-^ mice at P25 (Fig 6A), when the superficial, intermediate and outer vascular plexus vessels are fully mature. Isolectin B4 staining of retinas of *Cd34*^-/-^ mice revealed no abnormalities in development in any of the retinal vascular plexus layers.

Although we did not detect PODXL expression on tip cell filopodia of advancing retinal vessels in wild type mice, it is possible that, in the absence of CD34, PODXL is expressed as a compensatory mechanism. However, immunohistochemistry of whole-mount retinas revealed no difference in PODXL expression on the vasculature in the developing retinas of wild type and *Cd34*^-/-^ mice. Moreover PODXL staining was not detected in filopodia at the angiogenic front in *Cd34*^-/-^ mice (Fig 6B).

From this data we concluded that, although the CD34 enhances formation of filopodia at the angiogenic front of retinal vessels, CD34 is dispensable for vascularization of the mouse retina. In addition, PODXL does not compensate for loss of CD34 expression during retinal angiogenesis. These data suggest that CD34 expression marks endothelial tip cells during retinal angiogenesis, but it is not functionally required for vessel development in the retina.

### CD34 is involved in epi-retinal neovascularization but is dispensable for restoration of retinal vasculature following vaso-obliteration

We next used the OIR model to determine whether ablation of *Cd34* impairs pathological retinal neovascularization. The OIR model is an acute bi-phasic model of pre-retinal neovascularization associated with ischemia in the retina that develops after a period of experimental hyperoxia. In the first phase, P7 mouse pups are exposed to a high oxygen concentration (75% O_2_) for 5 days (P7-P12) to cause retinal vaso-obliteration in the central posterior retina [37]. In the second phase, pups are returned to normoxia where the relatively low oxygen concentration of 21% causes hypoxia and consequent excessive angiogenesis beyond the retina into the vitreous. Endothelial cell migration and invasion through the inner limiting membrane (the boundary between the neuroretina and the vitreous cavity) is an early phase of the formation of pathological neovascular tufts that expand beyond the retina in the OIR model.

**Fig 7:**
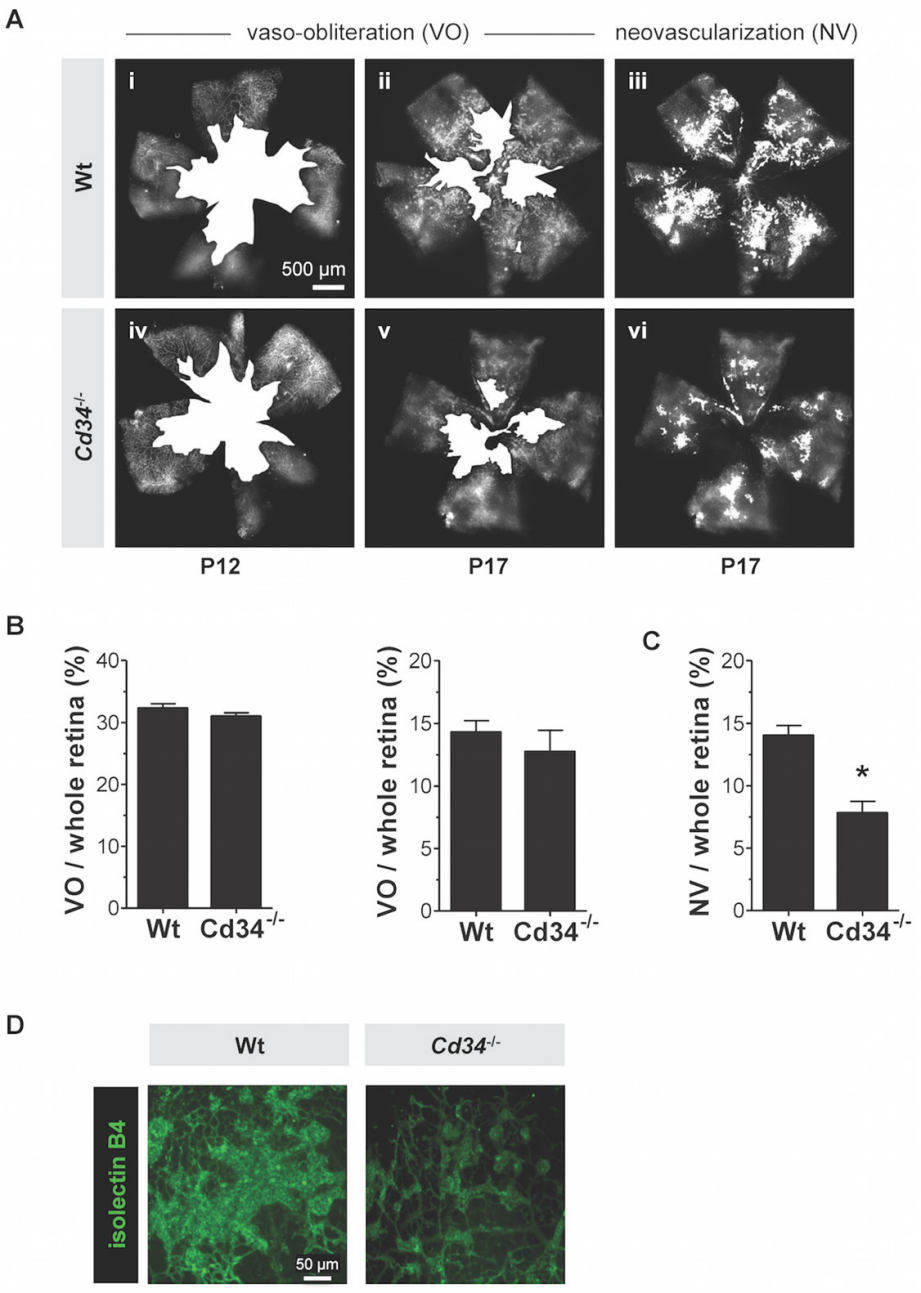
Loss of CD34 limits formation of pathological neovascularization in oxygen-induced retinopathy (OIR). (A) Representative retinal images showing total retinal area and regions of vaso-obliteration (VO) in OIR model for wild type (Wt) and *Cd34*^-/-^ mice at P12 and P17 and neovascularization (NV) at P17. Scale bar = 50 μm (B) Ratios of VO to total retinal area indicated no significant difference in VO between wild type and *Cd34*^-/-^ mice. (C) At P17, neovascularization was significantly decreased in *Cd34*^-/-^ mice as compared to wild type mice (*P < 0.01). Error bars represent standard deviation. * Significantly different from wild type with P < 0.05. (D) Isolectin B4 labeling of retinas harvested at P25 after OIR revealed that epi-retinal tufts in wild type mice aggregated as large continuous areas of neovascularization whereas in *Cd34*^-/-^ mice, the considerably smaller tufts did not aggregate. Scale bar = 50 μm.

We first analyzed the degree of vaso-obliteration in *Cd34*^-/-^ and wild type pups after 5 days of hyperoxia (P12) and found that exposure to hyperoxia resulted in a significantly reduced number of retinal capillaries especially in the central area (Fig 7A) but the extent of vaso-obliteration was identical in wild type and *Cd34*^-/-^ mice at P12 and P17 (Fig 7A and 7B). However, the ratio of the area of pre-retinal neovascularization to the total retinal area was significantly lower at P17 in *Cd34*^-/-^ mice (7.8%) as compared to wild type mice (14.0%) (Fig 7C). Furthermore, confocal images of P17 OIR retinas labeled with isolectin B4 revealed that epi-retinal tufts in wild type mice aggregated as large continuous areas of neovascularization whereas in *Cd34*^-/-^ mice, the considerably smaller tufts did not aggregate (Fig 7D). At P25, vaso-obliteration or neovascularization was not detected in OIR-treated wild type or *Cd34*^-/-^ mice and both genotypes appeared to have recovered the retinal vasculature at this stage (data not shown). Thus, in a model of pathological angiogenesis, the absence of CD34 reduces epi-retinal vessel tuft formation, a process that contributes to vision impairment in human retinopathies.

## Discussion

Earlier studies showed that CD34 is actively regulated on vascular endothelial cells and that angiogenic factors play a role in this regulation [7, 12, 13, 16, 21–23, 27, 38]. The role of VEGF in CD34 expression on endothelial cells *in vitro* has been controversial with studies showing that it either downregulates [12, 38] or upregulates [21, 27] expression. We hypothesized that the anti-adhesive function of CD34 is relevant in angiogenesis during invasion of endothelial tip cells through the basal lamina and other extracellular matrix structures. This hypothesis was derived from our previous discoveries that CD34 enhances adhesion, mobility and invasiveness of hematopoietic progenitors, mast cells, and eosinophils [17–20]. The observation of high expression of CD34 on endothelial tip cells and their filopodia suggests a similar molecular function [7, 13, 21–23]. Adhesive and anti-adhesive interactions between cell surface proteins and individual components of the vascular basal lamina determine the number and the behavior of endothelial tip cells during angiogenesis [4]. We showed that sprouting angiogenesis activity in HMEC-1 cultures is enhanced in the CD34^+^ fraction of HMEC-1 cells, and silencing of CD34 expression in HMEC-1 cells inhibits their response to exogenous VEGF-A. Migration is a key characteristic of tip cells. However, we did not find any effect of silencing of CD34 on HMEC-1 invasion or migration *in vitro*. We conclude that the absence of CD34 prevents VEGF-induced angiogenic sprouting *in vitro*, but does not limit endothelial cell migration or invasion *in vitro*.

During developmental angiogenesis in the mouse retina, CD34 is expressed on filopodia of endothelial tip cells in a distribution pattern similar to that of isolectin B4 [22]. We show that ablation of CD34 in mice reduces the formation of filopodia in retinal tip cells by approximately 30% during developmental retinal angiogenesis. However, this reduction in filopodia was not sufficient to cause a loss of vessel density in the fully developed retinas in *Cd34*^-/-^ mice. This is consistent with observations by Sawamiphak et al [39] who report that reduction of filopodial density by at least 50% is needed to attenuate vessel density in the retina and Phng et al who show that filopodia are dispensable for angiogenesis [8]. Because we have previously shown that PODXL is a potent inducer of microvillus formation in epithelial cells [40]and because microvilli and filopodia share many structural similarities and molecular components, we considered the possibility that PODXL compensates for the loss of CD34 in the formation of endothelial tip cell filopodia. However, we did not detect PODXL expression on filopodia at the retinal vessel front in either wild type or *Cd34*^-/-^ mice.

We found that CD34 did not impair physiological retinal angiogenesis aimed to restore the vasculature due to OIR. However, CD34 ablation impaired pathological epi-retinal tuft formation in the OIR model. Intriguingly, Budd et al. showed that reduced numbers of endothelial tip cell filopodia corresponded to a reduced intravitreal but not intra-retinal vascularization [41]. Therefore, it is possible that the diminished number of filopodia in retinal tip cells in *Cd34*^-/-^ mice hampered intravitreous invasion. This suggests that CD34 has a functional role in VEGF-mediated vessel growth.

## Conclusion

In conclusion, our study shows that CD34 is important for the formation of vascular sprouts and filopodia but CD34 does not appear to be essential for the development of an intact retinal vascular network. However, the absence of CD34 limits invasion of vessels into the vitreous and formation of epi-retinal tufts in the OIR model, which suggests that CD34 is involved in the pathological neovascularization causing vision loss in proliferative retinopathies.
