# Correction: CD34 Promotes Pathological Epi-Retinal Neovascularization in a Mouse Model of Oxygen-Induced Retinopathy

**DOI:** 10.1371/journal.pone.0169061

**Published:** 2016-12-21

**Authors:** Martin J. Siemerink, Michael R. Hughes, Marchien G. Dallinga, Tomek Gora, Jessica Cait, Ilse M. C. Vogels, Bahar Yetkin-Arik, Cornelis J. F. Van Noorden, Ingeborg Klaassen, Kelly M. McNagny, Reinier O. Schlingemann

The seventh author’s name is spelled incorrectly. The correct name is: Bahar Yetkin-Arik.
